# Mild to moderate partial sleep deprivation is associated with increased impulsivity and decreased positive affect in young adults

**DOI:** 10.1093/sleep/zsaa078

**Published:** 2020-04-19

**Authors:** Ingvild Saksvik-Lehouillier, Simen Berg Saksvik, Johanna Dahlberg, Tiril K Tanum, Heidi Ringen, Håvard Rudi Karlsen, Trine Smedbøl, Torhild Anita Sørengaard, Mailen Stople, Håvard Kallestad, Alexander Olsen

**Affiliations:** 1 Department of Psychology, Norwegian University of Science and Technology, Trondheim, Norway; 2 Department of Physical Medicine and Rehabilitation, St. Olavs Hospital, Trondheim University Hospital, Trondheim, Norway; 3 Department of Mental Health, Norwegian University of Science and Technology, Trondheim, Norway

**Keywords:** speed-accuracy trade-off, sleep restriction, short sleep, sleep deprivation, affect, cognitive control function, executive function

## Abstract

The effects of mild–moderate partial sleep deprivation on affective and cognitive functioning were evaluated in a naturalistic home environment, mimicking short sleep typically caused by demands from work or society. A total of 52 healthy individuals aged 18–35 was included in an 11-day study protocol. Participants slept at home, and sleep patterns were observed using actigraphs and sleep diaries. After maintaining habitual sleep for 7 days, the participants were asked to sleep 2 hours less than their average sleep duration for the last three nights of the study protocol. A not-X continuous performance test was administered at 9 am (± 90 minutes) on days 1, 4, 8 (habitual sleep), 9 and 11 (sleep deprivation). Performance-based measures included response accuracy and speed. Participant-reported measures included how well the participants felt they performed and how exhausted they were from taking the test, as well as positive and negative affect. There was a significant change in reaction time, number of commission errors, subjective performance, subjective exertion, and positive affect across the visits. Specifically, there was a linear decrease in reaction time, performance, and positive affect throughout the study, and a significant quadratic trend for commissions and exertion (first decreasing, then increasing after sleep deprivation). The univariate tests for omissions and negative affect were not significant. We conclude that sleeping 1.5–2 hours less than usual leads to faster response speed, but more commission errors and decreased positive affect. This indicates that individuals become more impulsive and experience less positive affect after a period of short sleep.

**Statement of Significance**

In this study, we asked participants to sleep 1.5–2 hours less than they usually do for three consecutive nights in their own home. We found that this individually calculated mild to moderate sleep deprivation changed several cognitive and affective processes, indicating that the subjects became more impulsive and experienced reduced positive affect in the morning after the sleep deprivation compared with normal sleep. With these findings, we show that the sleep loss many individuals experience during a normal week significantly affects morning cognitive and emotional functioning, which may increase the risk of mistakes and accidents in everyday life. This could limit the capacity to manage negative life events and stress. Future studies need to investigate individual differences in this change in cognition and affect.

## Introduction

Lack of sufficient sleep is associated with cognitive and emotional problems [1, 2] and an increased risk of accidents [3]. Despite the known negative effects of insufficient sleep, shorter sleep has become more common in the last 5 years among adults [4]. Seven to 9 hours of sleep is recommended for young adults and 7–8 hours for older adults [5]. Still, 29.2% of all adults in the 2012 US National Health Survey reported that they sleep less than 6 hours per night [6]. Prior studies have examined how partial sleep deprivation influences cognition and affect, but few have investigated mild–moderate partial sleep deprivation in a naturalistic setting [7]. Also, extant studies have not simultaneously assessed how sleep deprivation may influence both performance-based and participant-reported measures. Accordingly, we lack a broad picture of the effect of mild–moderate sleep deprivation on cognitive and affective processes.

Most research focuses on the effects of total sleep deprivation, while partial sleep deprivation is more common in everyday life [8]. Being deprived of sleep leads to several changes in brain function [9], and short-term total sleep deprivation typically shows negative effects across several cognitive domains [10]. Both total and partial sleep deprivation markedly affect an individual’s capacity to sustain attention and maintain vigilance [8, 11], especially for attention tasks with relatively simple task demands [10, 12]. Moreover, partial sleep deprivation, by restricting sleep to 5 hours per night, increases the number of lapses of attention and increases response speed after only two to three nights [13, 14]. A recent meta-analytical review shows that partial sleep deprivation can have negative effects on several cognitive domains, especially sustained attention and executive function [15]. Still, sleeping only 1 hour less than normal does not seem to influence sustained attention and response inhibition [7]. Thus, the critical limit for mild–moderate sleep deprivation remains to be determined.

Sleep loss and poor sleep quality negatively affect how the brain process emotions after a night of poor sleep [16]. Both the ability to express and regulate emotions are affected by lack of sleep [17]. Partial sleep deprivation measured in laboratory settings seems to be associated with decreased positive affect in adolescents and adults [18]. Some studies find no change in negative affect following sleep deprivation [18, 19], whereas others show that partial sleep deprivation may lead to worsening in mood or increase in negative affect [20, 21]. This indicates that sleep deprivation may lower the psychological threshold for experiencing stress and negative affect (i.e. lower cognitive control) in contexts with higher cognitive demands [22, 23]. However, the exact mechanisms of such alterations remain largely unknown, and there is a need for studies investigating both cognition and affect in a naturalistic context of mild–moderate sleep deprivation.

Studies investigating effects of partial sleep deprivation have typically been performed in a laboratory setting, but naturalistic actigraphy studies are recommended when investigating the effects of partial sleep deprivation on cognition to provide more naturalistic and ecologically valid effects [15]. Recently, some studies using this approach have been performed [7, 24], but a challenge with these studies is a lack of control over prior sleep and/or a too-short study period. Although naturalistic and ecologically valid studies are called for, it is still necessary to maintain as much control over the experimental setting as possible. It is therefore necessary for future research to perform naturalistic actigraphy studies of partial sleep deprivation with a higher level of control than what has been the case for previous studies. This should be done by controlling for sleep prior to the sleep deprivation with actigraphy and sleep diary, measuring partial sleep deprivation across several nights, and including more than one baseline test to control for practice effects. It is critical that most previous research performed on partial sleep deprivation has investigated the effects of sleeping a given number of hours, typically 4–5 hours, without considering individual sleep needs. This may lead to a more extensive sleep deprivation than people typically experience in daily life. Thus, the results may not be generalizable to the everyday mild–moderate sleep deprivation many people experience in today’s society.

The aim of this study was to incorporate a multiparametric perspective to investigate the effect of mild–moderate partial sleep deprivation on cognitive and affective processes experienced in the morning. To mimic naturalistic short sleep caused by demands from work or society in general, healthy young participants were observed in a naturalistic home environment, and the sleep deprivation protocol was adjusted for individual sleep needs. We hypothesized that mild–moderate partial sleep deprivation in a naturalistic setting would have negative impacts across several cognitive and affective domains measured in the morning.

## Methods

### Sample

**Figure 1.**
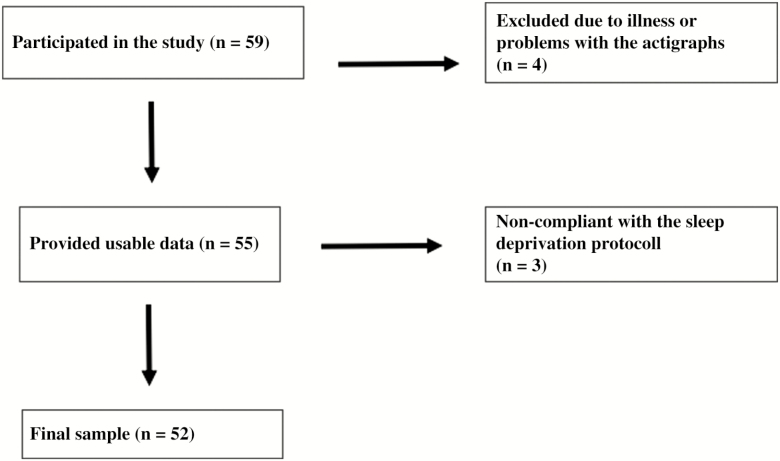
Flow chart of participants.

A total of 59 healthy individuals aged 18–35 participated in this study. Inclusion criteria were 18–35 years of age and fluency in the Norwegian language. Exclusion criteria were any self-reported severe psychiatric, neurological, or medical conditions. Apart from this, the participants prior sleep habits and sleep quality were not considered in the inclusion or exclusion criteria. Participants were recruited through ads at different university campuses and, nearby, through social media and in lectures. Figure 1 shows a flow chart of the recruitment process. Because we were interested in testing mild–moderate partial sleep deprivation (in line with ref. [15]), we decided to include all participants who successfully complied with the sleep restriction protocol by reducing their sleep for at least 90 minutes or more on all 3 days of the sleep deprivation condition. Seven participants were excluded from the final analyses because of illness during the study or problems with the actigraphs (*n* = 4) or because they could not comply with the sleep deprivation protocol (*n* = 3). The final sample included in the analyses comprised 52 individuals, 41 (78.8%) of these were women, and the mean age was 22.57 (*SD* = 3.09) years.

### Ethics

The study protocol was approved by the Regional Committee for Medical and Health Research Ethics in Central Norway (REK number 2017/85) and was in accordance with the 1964 Helsinki Declaration and its later amendments or comparable ethical standards. Written informed consent was obtained from all participants.

### Study design

**Figure 2.**
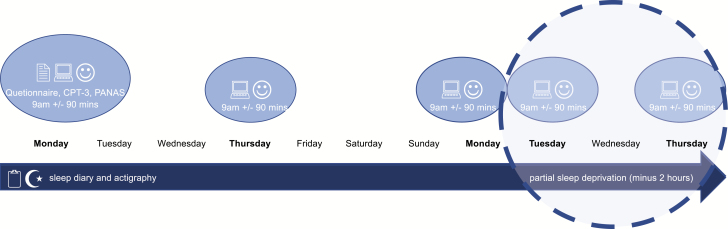
Flow chart of the procedure.

A within-group multiple baseline experimental design was applied (see flow chart in Figure 2).

### Procedure

The participants took part in an 11-day study protocol with partial sleep deprivation applied during the last 3 days of the study (Figure 1). In the mild–moderate [25] partial sleep deprivation protocol, participants were asked to sleep 2 hours less than their average sleep duration in the habitual sleep period (first seven nights). The participants were asked to go to bed 2 hours later than usual and get up at the same time in the morning as in the habitual sleep period. Cognitive and emotional function was assessed at five time points; three times during the habitual sleep period: visit 1 (V1), visit 2 (V2) and visit 3 (V3), and two times during the sleep-deprived condition: visit 4 (V4) and visit 5 (V5).

The participants were tested in groups of 3–15 individuals at different times of the year when the light conditions varied from sunrise at 04.58 am to 06.54 am and from sunset at 6.04 pm to 9.34 pm. Every data collection period started on the same day of the week (Monday). All participants had to meet for five visits on five different days (Monday week 1, Thursday week 1, Monday week 2, Tuesday week 2, and Thursday week 2), every time in the same time slot (see flow chart). Participants were tested at 09.00 in the morning, ± 90 minutes, in line with other experimental sleep deprivation studies [26]. All participants were asked to not consume any caffeinated drinks between awakening and testing.

### Instruments

All participants completed a baseline questionnaire, including demographic information and a range of established and validated instruments measuring sleep, emotional functioning, fatigue, pain, cognitive functioning, and individual differences. The following instruments assessed sleep and sleepiness: Insomnia Severity Index [25], Pittsburgh Sleep Quality Index [27], and Epworth Sleepiness Scale [28], in addition to single questions used in epidemiological studies on sleep duration. The Hospital Anxiety and Depression Scale was included to measure anxiety and depression [29].We used the Fatigue Severity Scale to measure fatigue [30] and the Diurnal Scale to measure morningness/eveningness [31].

All participants were asked to complete a sleep diary every morning during the study. The sleep diary was a modified version of the diary published by Morin [32] and included questions about bedtimes, rise times, sleep latency, and wake periods in the night, enabling us to calculate the participants’ subjective sleep duration and sleep efficiency. In addition, the sleep diary included questions about naps, daytime sleepiness, and subjective sleep quality. Subjective sleep quality was measured with one question asking the participants to rate the quality of sleep on a scale from 1 (= very light) to 5 (=very deep) for each day.

#### Actigraphy

Participants were asked to wear a wrist-worn actigraph device for the whole study period (Actiwatch Spectrum Pro, Philips Respironics, USA). In addition to activity measures based on an accelerometer, this device recorded time and date indicators, event markers, and illuminance monitoring. The actigraphs collected data in 15-second epochs. The actigraphs were used to assess the participants’ total sleep time, sleep efficiency, and time of bedtime and rise time (including midpoint of sleep). We used the actigraphy data collected during the habitual sleep period to calculate the participants’ individual total sleep time in the sleep-deprived condition and manually cross-checked and adjusted bed time and rise time based on sleep diary data [33], as well as systematic inspection of the automatically coded rest periods in the actograms based on activity, light conditions, and event markers [34]. The participant´s mean total sleep time in the habitual sleep period was assessed in the actigraphy software on site (Philips Actiware 6.0.0), and the shortened sleep time was conveyed to the participants verbally and in writing. Actigraphy total sleep time minimum, maximum, mean and standard deviations can be seen in **Table 1**.

**Table 1.** Baseline measures of cognition, affect, sleep, mental health, diurnal preference, and demographic characteristics of the sample measured at visit 1 (*n* = 47–52)

	Mean (*SD*)
Gender	41 (78.8%), females
Age	22.58 (3.06)
Commissions	52.00 (8.87)
Omissions	46.15 (2.50)
Reaction time	41.67 (5.04)
Negative affect	13.86 (3.75)
Positive affect	26.82 (5.81)
Performance	4.71 (1.31)
Exertion	4.83 (1.92)
Insomnia^a^	5.77 (3.65)
Sleep quality^b^	3.25 (2.05)
Sleepiness^c^	7.51 (4.48)
Anxiety^d^	5.56 (3.38)
Depression^d^	2.82 (2.42)
Fatigue^e^	3.9 (1.01)
Diurnal preference^f^	17.38 (4.37)

^a^Measured with Insomnia Severity Index.

^b^Measured with Pittsburgh Sleep Quality Index, where low score indicates good sleep quality.

^c^Measured with Epworth Sleepiness Scale.

^d^Measued with Hospital Anxiety and Depression Scale.

^e^Measured with Fatigue Severity Scale.

^f^Measured with Diurnal Scale.

#### Conners’ Continuous Performance Test-3

The Conners’ Continuous Performance Test (CCPT) [35] is an extensively used and well-validated not-X continuous performance test that was used to assess performance-based cognitive control function. Letters A–Z are consecutively presented on the screen in a pseudorandom fashion for 360 trials with a duration of 14 minutes. The participants were instructed to press a button each time a letter is presented on the screen, except for the letter X. Both response speed (hit reaction time, the mean response speed, measured in milliseconds, for all correct responses to target made during the test) and accuracy (omission errors and commission errors) were extracted and used in analyses. Immediately after the test, participants were asked to rate their perceived *performance* and *exertion* on a scale from 1 to 10 (very bad—very good performance; no exertion at all—very much exertion).

#### The Positive and Negative Affect Schedule

The Positive and Negative Affect Schedule (PANAS) was used as a self-report measure of positive and negative affect. Positive affect and negative affect reflect independent (orthogonal) affective state dimensions [36]. The scale consists of 20 items (descriptors) describing various feelings and emotions. Respectively, 10 scale items correspond to positive affect (e.g. excited, determined, alert) and 10 items to negative affect (e.g. fear, guilt, nervousness). Cronbach’s alpha for positive affect and negative affect at baseline was acceptable at .77 and .75.

### Statistical analysis

Paired-sample *t*-tests were used to evaluate differences in sleep duration and sleep efficiency/quality measured with actigraphy and the sleep diary, midpoint of sleep measured with actigraphy, as well as differences between the habitual sleep period and the sleep-deprived condition. We considered cognitive function, affect, and self-reported performance and exertion to represent three a priori different domains to be evaluated. Separate repeated-measures analyses of variance (rmANOVAs) were therefore performed to investigate the effects of cognitive function, affect, and self-reported measures of performance and exertion throughout the study. Each domain had two or more submeasures and we therefore chose to test these in the same models. Our data analysis strategy was mainly motivated by three important features of the rmANOVA in the context of complete data from all time points: (1) the opportunity of evaluating polynomial trends potentially associated with learning effects, and/or dose–response relationships; (2) the opportunity of delineating interaction effects (e.g. speed-accuracy trade-off, positive-negative affect interaction, etc.); and (3) the powerful but reasonable control for multiple comparisons provided by the ANOVA. To investigate cognitive functioning across the different time points, we performed a 3 × 5 rmANOVA with cognitive functioning (hit reaction time, commission errors, and omission errors) as the dependent variable and time (V1, V2, V3, V4, and V5) as a fixed factor. To investigate self-reported measures across the different time points (self-reported *exertion* and *performance* on the Conners’ Continuous Performance Test-3 [CCPT-3] test), we performed a 2 × 5 rANOVA with self-reported measures (*performance* and *exertion*) as the dependent variable, and time (V1, V2, V3, V4, and V5) as a fixed factor. To investigate affect across the different time points, we performed a 2 × 5 rANOVA with affect (*positive* and *negative affect*) as the dependent variable and time as a fixed factor. For all rANOVAs, we tested the assumption of sphericity using Mauchley’s test. If the assumption was violated, the following *F* tests were corrected using the Greenhouse–Geisser (ε) method [37]. In case of significant main or interaction effects, we performed univariate analyses and polynomial trend analyses to further break down the specific effects. *P*-values < .05 were considered statistically significant. Partial eta squared (η _p_^2^) was used as a measure of effect size. All analyses were performed in SPSS v.25.

## Results

In Table 1, we report baseline means and standard deviations for demographics, sleep, and health information of the participants. The means indicate that the group was relatively healthy, as illustrated in, for example, the mean scores of anxiety and depression, which were lower than a normative sample of the general UK population [29]. Moreover, the scores for sleep quality were better; scores for fatigue were lower, and scores for insomnia were similar to the means of a US college student sample reported in an epidemiological study of sleep among students [38].

Table 2 shows sleep duration, sleep efficiency, midpoint of sleep, and subjective sleep quality for all participants represented in mean scores across the habitual sleep period and across the three days of partial sleep deprivation. Sleep duration was statistically significantly shorter in the sleep-deprived condition compared to the habitual sleep period (*t* = 34.21, *p* < .001). The participants slept for an average of 124 minutes (slightly over 2 hours) less during the sleep deprived condition compared with the habitual sleep period. Sleep efficiency was higher during the habitual sleep period compared with the partial sleep deprivation period. There were no statistical differences in the midpoint of sleep in the two conditions. Subjective sleep quality was higher during sleep-deprived condition compared with the habitual sleep period.

**Table 2.** Means and *SD* for sleep measures during the 7 days of habitual sleep period and during the 3 days of partial sleep deprivation (*n* = 49–52)

	Habitual sleep period			Sleep deprived				
	Min	Max	Mean (*SD*)	Min	Max	Mean (*SD*)	*t*	*p*
Sleep duration actigraphy (min)	327	517	435 (41)	214	382	301 (43)	38.09	.000
Sleep duration sleep diary (min)	360	553	452 (42)	217	392	312 (43)	34.21	.000
Sleep efficiency actigraphy (%)	76.3	93.5	86.9 (3.7)	68.5	97.1	86.9 (5.3)	−0.12	.906
Midpoint of sleep actigraphy (time)	2.57 am	6.21 am	4.22 am (00:49 min)	3.01 am	6.53 am	4.26 am (00:51 min)	−1.43	.159
Subjective sleep quality (1–5)	2.3	4.7	3.5 (0.6)	2.7	5.0	4.1 (0.7)	−6.26	.000

### Cognitive function, self-reported measures, and affect in habitual sleep and partial sleep deprivation

**Figure 3.**
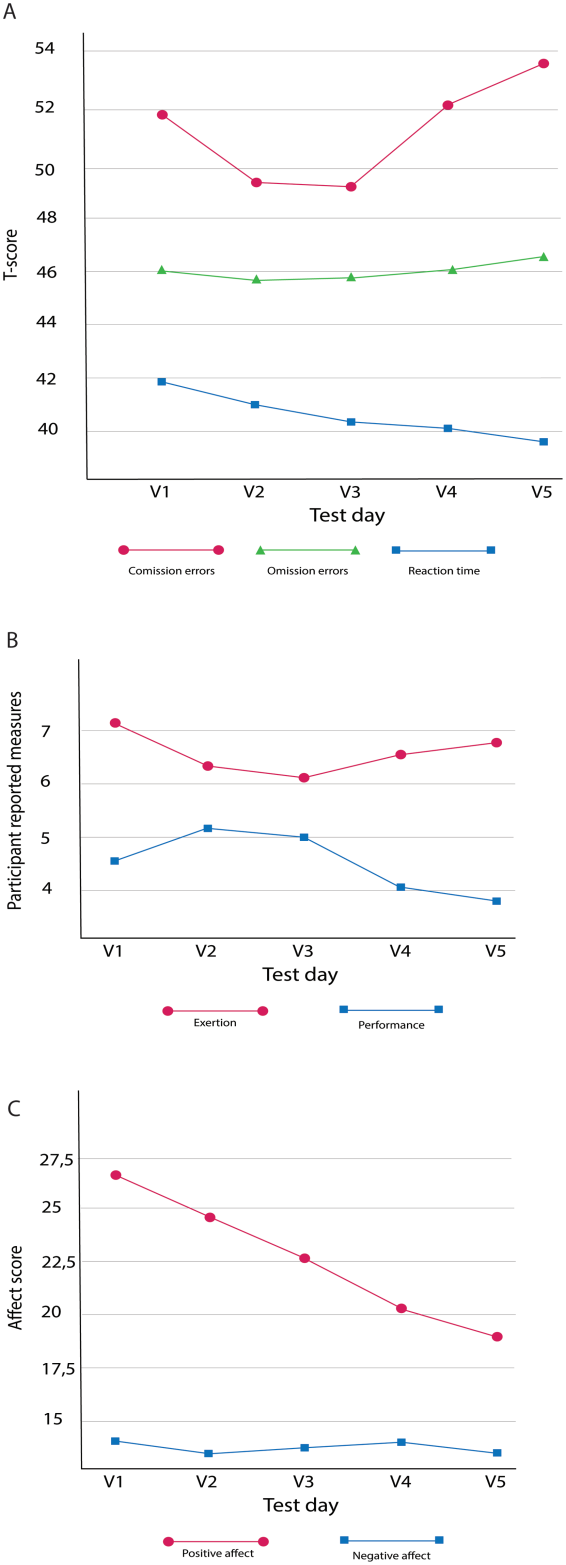
Change in cognition (A), self-reported measures (B), and affect (C) over time.

Table 3 shows the mean scores at all five measure points on commissions, omissions, hit reaction time, subjective performance, subjective exertion, negative and positive affect, as well as the results from the analyses. The mean differences are also illustrated in Figure 3A–C.

**Table 3.** *F* values with corresponding degrees of freedom for analyses of changes in cognition, self-reported measures, and affect at the five visits, interaction effects of time and the different outcomes, as well as means and *SD* for all outcome variables

Outcome	Time	Time × outcome^a^	Mean (*SD*) V1		Mean (*SD*) V2		Mean (*SD*) V3		Mean (*SD*) V4		Mean (*SD*) V5	
Cognition	28.35** (1,6)	3.82* (4,185)										
Reaction time	10.05** (4,188)		41.73	(4.84)	40.73	(5.15)	40.19	(5.43)	39.94	(5.37)	39.25	(5.30)
Commission errors	7.12* (3,152)		52.02	(9.05)	49.58	(9.47)	49.46	(10.58)	52.15	(10.82)	53.35	(11.98)
Omission errors	1.29 (2,79)		46.12	(2.47)	45.38	(1.47)	45.46	(1.71)	46.15	(6.58)	47.29	(8.79)
Self-reported measures	45.05** (1,47)	8.41** (4,188)										
Performance	7.08** (4,188)		4.67	(1.26)	5.13	(1.71)	5.00	(1.90)	4.06	(1.60)	4.00	(1.74)
Exertion	3.79* (3,135)		7.00	(1.74)	6.27	(2.34)	6.08	(2.04)	6.50	(2.06)	6.60	(2.21)
Affect	122.99** (1,48)	14.14** (3,162)										
Negative affect	0.55 (3,141)		14.16	(3.85)	13.61	(3.63)	13.88	(4.15)	14.06	(4.05)	13.59	(2.96)
Positive affect	26.37** (4,192)		26.84	(5.92)	24.45	(7.50)	22.57	(6.01)	20.14	(6.04)	18.90	(6.03)

V1–V3 = baseline, V4–V5 = sleep deprived.

^a^Outcome is cognition, affect, or self-reported measures.

*P < .05; **p < .001.

The assumption of sphericity was violated for *cognition*, *commission errors*, *omission errors*, self-reported *exertion*, and *negative affect*. For these variables, the *F* tests were therefore corrected with the Greenhouse-Geisser (ε) method for the main effect of cognition ε = .65, commission errors ε = .81, omission errors ε = .42, self-reported exertion ε = .72, and negative affect ε = .738.

### Changes in cognition after partial sleep deprivation

There was a statistically significant main effect of cognition *F* (1, 61) = 28.35, *p* < .001, η _p_^2^ = .376. Univariate tests showed a significant effect for hit reaction time, *F* (4, 188) = 10.05, *p* < .001, η _p_^2^ = .18 and commission errors, *F* (3, 152) = 7.12, *p* < .001, η _p_^2^ = .13. There was no significant effect for omission errors *F* (2, 79) = 1.29, *p* = .227, η _p_^2^ = .027. A polynomial trend analysis revealed that hit reaction time decreased linearly throughout the five study visits (*p* < .001, η _p_^2^ = .341 (see Figure 3A, blue line), including both the habitual sleep period and the sleep-deprived condition. Commission errors showed a significant quadratic trend between visits, *p* < .001, η _p_^2^ = .238 (see Figure 3A, red line). As illustrated in Figure 3A, the quadratic trend implies that the changes in commission first decrease and then increase, and the change from decrease to increase occurs after visit #3 when the sleep deprivation is implemented.

Moreover, there was a significant interaction effect between the dependent variable cognition and the fixed factor time *F* (4, 185) = 3.82, *p* = .005, η _p_^2^ = .075. The contrast showed a significant linear interaction effect between hit reaction time and commission errors *F* (1, 47) = 13.52, *p* = .001, η _p_^2^ = .221, as well as a significant linear interaction effect between hit reaction time and omission errors *F* (1, 47) = 5.69, *p* = .021, η _p_^2^ = .108. Figure 3A shows that as the hit reaction time decreased, the number of omission errors and commission errors increased relative to the changes in hit reaction time. There was no significant interaction effect between commission and omission errors.

### Changes in self-reported measures after partial sleep deprivation

Results from the 2 × 5 rANOVA showed a main effect of *self-reported measures*, *F* (1, 47) = 45.05, *p* < .001, η _p_^2^ = .489. Univariate tests showed significant effects for self-reported performance, *F* (4, 188) = 7.08, *p* < .001, η _p_^2^ = .131 and self-reported exertion between visits, *F* (3, 135) = 3.79, *p* = .013, η _p_^2^ = .075. Self-reported performance decreased linearly across visits (*p* = .001, η _p_^2^ = .235) (see Figure 3C, blue line). Self-reported exertion first decreased during the habitual sleep period (three first visits) of the study and then increased throughout the partial sleep deprivation period, creating a quadratic curve, *p* < .001, η _p_^2^ = .24 (see Figure 3C, red line).

There was also a significant interaction effect between the dependent variable self-reported measures and the fixed factor time, *F* (4, 188) = 8,41, *p* < .001, η _p_^2^ = .152. The contrast showed a significant quadratic interaction effect between self-reported performance and self-reported exertion, *F* (1, 47) = 23.71, *p* < .001, η _p_^2^ = .335. This quadratic interaction demonstrates that compared with self-reported exertion, self-reported performance increased during the first visits of the study and then decreased, while the opposite trend was seen for self-reported exertion (Figure 3C).

### Changes in affect after partial sleep deprivation

The 2 × 5 rANOVA showed a main effect of the dependent variable affect, *F* (1, 48) = 122.99, *p* < .001, η _p_^2^ = .719. Univariate tests showed significant changes in positive affect across the different visits, *F* (4, 192) = 26.37, *p* < .001, η _p_^2^ = .355 (see Figure 3B, red line). Negative affect did not differ significantly between the visits, *F* (3, 141) = 0.55, *p* = .648, η _p_^2^ = .011. Positive affect decreased linearly across the five visits, *p* < .001, η _p_^2^ = .680.

Lastly, the analyses showed a significant interaction effect between the dependent variable affect and the fixed factor time, *F* (3, 162) = 14.14, *p* < .001, η _p_^2^ = .228, and the contrast showed a significant linear interaction effect between positive and negative affect over time, *F* (1, 48) = 52.70, *p* < .001, η _p_^2^ = .523. This indicates that relative to negative affect, positive affect decreased linearly during the study period.

## Discussion

The present study demonstrates that sleeping 1.5–2 hours less than usual per night for 1–3 days in a home environment is associated with poorer cognitive control function in the morning, as reflected by increased impulsivity (faster hit reaction time but more commission errors), more exertion, poorer subjective performance, and decreased positive affect. This indicates that individuals become more impulsive, tired, and emotionally blunted after a period of short sleep. These effects were already present after 1 day of partial sleep deprivation and were further amplified throughout the next 2 days, indicating a dose–response relationship. These findings show that the sleep loss many adults experience in everyday life [6] may have detrimental effects on self-reported and performance-based cognitive performance and affect, which may have important implications for their health, productivity, and accident risk. Especially these effects may have severe consequences in the morning, on the way to work and in the beginning of the work day, possibly caused by sleep inertia [39].

### Increased impulsivity and exertion, poorer subjective performance, and decreased positive affect

Overall, our findings support studies indicating that mild–moderate partial sleep deprivation limits access to affective and cognitive resources [40]. We found that even though the participants reported putting more effort (exertion) into the CCPT-3 after sleep deprivation, they also reported performing worse compared with the baseline measures. This indicates that the participants tried to compensate for the effects of sleep loss with an increase in exertion [40]. Despite this increase in exertion, the participants had a decrease in both subjective and objective performance, as well as in positive affect. Thus, the participants in our study were aware of their reduced performance level. Compensatory exertion associated with only partial recovery of performance has also been observed in a number of studies on total sleep deprivation [9]. Our study indicates that these compensatory efforts also occur after partial sleep deprivation. Importantly, previous studies have shown that chronic sleep restriction is associated with less awareness of the effects following sleep deprivation, when objective measures are used [41]. This might indicate that the awareness of ones’ performance may be reduced upon repeated nights of sleep deprivation. These findings may have important implications for everyday factors (such as work–life, driving, and social interactions) because the effects of sleep loss might not be reversed or withheld with increased exertion. Conversely, it is also possible that reduced positive affect (emotional blunting) leads to the observed impulsive behavior (faster hit reaction time and more errors) on the CCPT-3, as well as the reduced subjective performance and increased exertion after sleep deprivation. Reduced positive affect may lead to an increased feeling of poor performance and exertion performance on the test. It is possible that such reduced access to cognitive and affective recourses due to lack of sleep could lead to increased vulnerability for developing mental disorders. It is well-known that sleep problems are an important mechanism causing and maintaining several mental disorders [42].

Subjective sleep quality measured with sleep diary was higher during the sleep deprivation period compared with the habitual sleep period. Thus, despite the negative effects of sleep deprivation on subjective and objective measures of cognitive control and affect, the participants still reported sleeping better when sleep deprived than when they had normal sleep. This is in line with previous studies reporting that partial sleep deprivation increases subjective sleep quality [43]. During the sleep deprivation phase, participants were awake for longer during the evening and therefore had a possibility of longer exposure to light which may cause an underlying phase delay in the circadian rhythm relative to being in darkness [44]. However, there is also evidence that a high homeostatic sleep drive may reduce the phase-shifting capacity of light [45]. We did not include measures of circadian rhythm such as Dim Light Melatonin Onset in this trial. However, the sleep midpoint did not differ in habitual sleep compared with sleep deprivation, indicating that there was no major shift in the timing of the sleep–wake phase.

In our study, the tests were performed in the morning between 7.30 and 10.30 am. Laboratory studies have reported that cognitive performance is severely affected by sleep loss, especially in the morning [39], that sleep deprivation increase sleep inertia [46], and that sleep inertia can last up to four hours for some individuals [47]. Moreover, naturalistic studies have shown that sleep inertia can be present up to 2 hours after awakening from normal sleep length in a home environment [48]. Our participants were healthy young adults who had to travel to the university campus before testing, and hence, even though the partial sleep deprivation may have increased their sleep inertia, it is not likely that the participants still was in a state of sleep inertia at the time of testing.

### Cognitive control functioning

Our findings that partial sleep deprivation influences the number of errors and hit reaction time is in line with previous studies on sleep restriction [14], and total sleep deprivation [10], and the conclusions from a recent meta-analysis of a large amount of research in this topic [15]. However, some previous studies report an increase in response time following sleep deprivation [11, 14] and not a reduction as we found. The task in this research was the Psychomotor Vigilance Task (PVT). The PVT is one of the most-used cognitive tasks in sleep deprivation research and has provided valuable information for the field. However, one limitation with this test is that participants are asked to respond to relatively few and infrequent stimuli (targets). Although this has some advantages for detecting attentional lapses (omissions), this task, by design, provides rather unreliable response time estimates. In contrast, the CCPT-3 provides robust response time measures based on calculations including several hundred trials.

By indicating faster performance in speed and a decline in accuracy, our results support a speed-accuracy trade-off in line with what has previously been suggested by Lim and Dinges [10]. That is, faster responses may lead to a decline in accuracy, and vice versa.

We found that the number of errors and hit reaction time measured with CCPT-3 decreased at the three baseline measures, but the changes in hit reaction time and errors from the habitual sleep period to the sleep-deprived condition interacted. This shows that, in the sleep-deprived condition, as hit reaction time decreases, the number of errors increases. This is in line with prior findings of improved CCPT-3 performance in nonsleep-deprived participants [7]. Notably, however, in this study there were no changes in CCPT-3 performance after mild (1 hour) partial sleep deprivation over six consecutive days. Considering this finding, our results indicate that the negative effects of partial sleep deprivation on cognition may commence after sleep deprivation after close to 2 hours of deprivation. The effects of sleep deprivation in the study by Santisteban et al. [7] might also be masked by practice effects, as they only included one baseline measure and one sleep-deprived measure. It should also be noted that the participants in our study were rather high functioning, as they performed average or above average on all cognitive control variables at baseline. For example, at visit 3 (before sleep deprivation), the group mean score on hit reaction time was nearly 1 *SD* faster than the normative data provided with the CCPT, while at the same time, the number of commission errors was around the norm average. Also, the participants in our study had better scores on several baseline measures of health and sleep compared to norm samples [29] and comparable student samples [38].

Furthermore, the effect of sleep deprivation seemed to be dose dependent in our study, indicating that changes in emotional and cognitive functioning are more evident after several consecutive nights of sleep deprivation. Still, there are likely individual differences regarding sleep preference, resilience, habitual sleep duration, and other more stable dimensions at play in determining individual variation in vulnerability to mild–moderate sleep deprivation.

### Positive and negative affect

We observed a decrease in positive affect following sleep deprivation, but no change in negative affect after the sleep deprivation, which is in line with earlier laboratory findings [18, 19, 49]. In the present study, effects similar to those observed in earlier studies were revealed using a less-strict sleep restriction protocol. We also deployed individualized sleep deprivation time. This is important, as it suggests that small deviations from average total sleep time over consecutive days can cause a pronounced decline in experienced positive affect. We also observed that positive affect decreased at all the measurement times, also before the sleep deprivation, which may indicate that the effects were caused by being a part of the experiment (e.g. being less engaged upon repeated testing), and not merely the effects of partial sleep deprivation.

While our results are in line with other studies applying PANAS [18], they are somewhat in contrast to other previous studies reporting an increase in negative affect after sleep deprivation measured by Profile of Mood States (POMS) [13, 20, 21]. These two self-report measures therefore seem to capture different aspects of affective alteration following sleep deprivation. PANAS is designed to capture the presence or absence of active affects, and positive and negative affect are two distinct dimensions [36]. POMS assesses six dimensions of mood, five negative and one positive, which can be computed to one total mood disturbance score [50]. In our results, there was an interaction between positive and negative affect between visits. This can reflect distinct differences in how sleep deprivation influences positive and negative affect. Moreover, this demonstrates the importance of differentiating between positive and negative dimensions of affect in sleep deprivation studies. Altogether, our results lend support to studies indicating that the effect of sleep deprivation on negative affect is more dependent on the context than what is the case for positive affect [22, 23]. One night of total sleep deprivation leads to increased negative affect when the participants were exposed to a mild cognitive performance stressor [23]. However, in our study, we measured affect after performing the CCPT-3, which, for some, may also count as a mild cognitive performance stressor, and the reason we could not detect changes in negative affect after partial sleep deprivation in line with [23] may be due to differences in amount of sleep deprivation in our study compared to in [23], or because participants did not experience the CCPT-3 as a stressor because they are used to the test after taking it several times.

### Strengths, limitations, and suggestions for future research

The main strength of this study is the comprehensive design, including repeated measures using a combination of well-validated self-report, performance-based, and wearable sensor measures to study the effects of partial sleep deprivation in a naturalistic setting. Our study is the first to use a multiple baseline design; hence, we could use the participants as their own control. Moreover, our study is one of the few to investigate cognitive control function, subjective performance, exhaustion, and affect in the same study. A limitation with the present study is that the sleep deprivation condition was introduced at the same time in the protocol for everyone. One alternative approach could have been to use a crossover condition, where some participants had sleep deprivation at the beginning of the study period, while others experienced it at the end of the study period. This would provide us with different opportunities to adjust for order/practice effects. However, a disadvantage of crossover designs is that it is hard to estimate potential carryover effects. Importantly, as we tested the participants on three occasions (within-subject multiple baseline) before the sleep deprivation, we did have considerable control of the variance linked re-testing in our statistical analyses.

The study has a modest sample size, but still, our sample exceeds that of several previous similar studies [51–53]. Our sample comprises of mainly women (79%). Gender differences are therefore important to consider when interpreting interpretating our findings. Women seem to have higher vulnerability to both circadian and homeostatic influences following sleep deprivation compared to men measured in a laboratory setting [54]. In addition, women seem to experience more sleep difficulties, as well as more time awake during the night, and poorer sleep quality measured subjectively and with actigraphy compared to men [55], but still have better objectively measured sleep quality than men measured with polysomniography [56]. However, both sexes were included, and the distribution represents the sex distribution in many student groups cross-nationally.

## Conclusion

Short sleep duration is common in the adult population. We found that sleeping 1.5–2 hours less than usual for 3 days in a row in a home environment led to poorer cognitive control function measured at our lab in the morning. It also led to more exertion, poorer subjective performance, and reduced positive affect. These findings indicate that individuals become more impulsive after a period of short sleep. Moreover, we found that the participants had an increase in performance from the first baseline test to the third baseline test, but that 1 day of sleep deprivation overshadowed this effect, and the performance was further deteriorated after three nights of sleep deprivation. These findings highlight that even 1–2 hours less sleep for a few nights is associated with negative consequences. Also, these findings show that even a small lack of sleep may have important implications for everyday function and quality of life, such as social interaction, work efficiency, and traffic safety, especially in the early morning. Future research should focus on how such effects may vary with time of day and across different populations. It would also be interesting to see studies investigating how these effects can be remediated by psychological or medical interventions. Finally, we need knowledge determining the long-term accumulated effects and underlying biological mechanisms of mild–moderate partial sleep deprivation.
